# Characteristics of Individuals in Japan Who Regularly Manage Their Oral Health by Having a Family Dentist: A Nationwide Cross-Sectional Web-Based Survey

**DOI:** 10.3390/ijerph191710479

**Published:** 2022-08-23

**Authors:** Katsuo Oshima, Hiroko Miura, Rumi Tano, Hideki Fukuda

**Affiliations:** 1 Department of Dental Technology, The Nippon Dental University College, Tokyo 102-8159, Japan; 2 Division of Disease Control and Epidemiology, School of Dentistry, Health Sciences University of Hokkaido, Tobetsu 061-0293, Hokkaido, Japan; 3 Department of Health Promotion, National Institute of Public Health, Saitama 351-0197, Japan; 4 National Institute of Public Health, Saitama 351-0197, Japan

**Keywords:** family dentist, oral health, health policy, dental visits, web-based survey, Japan, public insurance system, socioeconomic factors

## Abstract

Dental healthcare systems may differ between countries; however, having a family dentist is generally important for proper oral health management. This study aims to analyze the proportion of people in Japan who have a family dentist, and their characteristics. A nationwide web-based survey with 3556 participants (1708 men and 1848 women) showed that 45.6% of men and 54.1% of women had a family dentist (FD group). A multiple logistic regression analysis revealed that men in the FD group mostly belonged to older age groups (≥70 s, OR: 2.41), received higher household incomes (≥8000 K JPY, OR: 1.47), brushed their teeth three or more times daily (OR: 1.60), practiced habitual interdental cleaning (OR: 3.66), and fewer lived in rural areas (towns and villages, OR: 0.52). Regarding the women, the majority belonged to older age groups (60 s, OR: 1.52; ≥70 s, OR: 1.73), practiced habitual interdental cleaning (OR: 3.68), and fewer received lower household incomes (<2000 K JPY, OR: 0.61). These results suggest that despite Japan being a country with a public insurance coverage system for both men and women, having a family dentist is associated with disparities in individual socioeconomic factors, particularly age and household income.

## 1. Introduction

It is important to manage oral health status and prevent dental diseases, such as dental caries and periodontal disease, through regular dental visits rather than visiting after symptoms develop, such as tooth pain and swelling. Such health behaviors, in addition to preventing tooth loss and protecting oral health, improve the quality of life [1,2,3].

Generally, regular dental visits are influenced by the healthcare systems of the respective countries. In Australia, Canada, and the United States, private health insurance plays an important role in financing dental care, suggesting that those who have dental insurance may perform more regular dental visits and have more preferable service utilization patterns than those without the insurance [4,5,6,7]. The system of financing dental care in developing countries does not protect the people from the economic consequences of oral conditions, and it is suggested that individuals’ out-of-pocket expenditures for dental care contribute to healthcare spending that can push households into poverty [8]. In 2010, the World Health Organization (WHO) addressed universal health coverage (UHC) in the WHO’s World Health Report [9]. Moreover, the United Nations (UN) adopted UHC as one of the Sustainable Development Goals (SDGs) in 2015 [10].

The Japanese healthcare system has adopted a scheme, in which all the citizens are covered by public insurance; this system covers both medical and dental care [11]. Most dental care costs are covered by public insurance and are set at an official price, except for some certain treatments, such as orthodontic treatment and dental implants. Patients pay a co-payment ranging from 10% to 30%, based on age and income. Furthermore, patients are free to access dental clinics anywhere, regardless of their personal conditions, the place they live, or the type of public insurance they have. As of 2020, there are 67,874 dental clinics in Japan, of which 97.7% advocate general dentistry [12], that is, these dental clinics can be described as having a primary-care function.

Considering these conditions of the Japanese healthcare system, Japanese citizens seem to be in a suitable environment for regular dental visits. Nevertheless, the importance of preventive dentistry has not been fully recognized in Japan, and an increasing number of people receiving regular dental visits is one of the challenges faced by the Japanese government [13]. According to the Japanese government’s Health Japan 21 (the second term) plan [13], the percentage of people who participate in dental checkups was set at 65% by 2022, compared to the base value of 34.1% in 2009. However, this target value has not yet been achieved (the latest measured value was 52.9%, as of 2016).

In Japan, there is no registration system of family dentists, whose purpose is to protect oral health through regular dental visits. Hence, the Japan Dental Association is enlightening the Japanese people to have a family dentist with whom they can consult for any oral health issues [14]. It has been reported that the system and ratio of having a family dentist varies greatly from country to country [15,16,17,18,19,20,21,22,23,24]; however, generally, having a family dentist is associated with maintaining good oral health status [16,19,23]. Furthermore, the studies conducted in this country showing the percentage and characteristics of those having a family dentist in certain local regions exist [16,17,18,19]; however, few studies have analyzed them on a nationwide scale.

Thus, we conduct an analysis to evaluate the actual status of family dentists in Japan, using a nationwide web-based survey. The aim of our study is to assess the actual conditions and individual characteristics of people who regularly manage their oral health by having a family dentist. Our results will help to clarify the challenges of having a family dentist in countries with a public insurance system, and present the data that will contribute to dental health policy planning.

## 2. Materials and Methods

### 2.1. Study Participants

This cross-sectional study obtained data from a web-based survey conducted over three days (from 6 to 8 September 2021). The survey was conducted by Macromill, Inc. (Tokyo, Japan) [25], a research company specializing in web-based surveys, on a registered pool of approximately 1.3 million people living in Japan. We set age as a criterion for study participants and included those who were 20 years of age and older. A total of 3556 study participants were randomly selected from the survey company’s database of registered individuals, using the quota sampling method. The sample distribution across genders (men and women), age groups (20 s, 30 s, 40 s, 50 s, 60 s, and 70 s and above), and regional blocks (Hokkaido, Tohoku, Kanto, Chubu, Kinki, Chugoku, Shikoku, and Kyushu regions) were considered to be representative of the Japanese population. This distribution was based on statistics obtained from the National Population Census of the Ministry of Internal Affairs and Communications [26].

The participants read an explanation of the purpose of the survey on the website and answered the questions after agreeing to participate. They received point-based incentives that could be converted to cash.

### 2.2. Outcome Variable

With respect to the outcome variable, we set up whether or not the survey participants were those who regularly managed their oral health condition by having a family dentist. Hence, based on the previous reports [14,27,28,29], in order to set this outcome variable, we defined the following as the condition of having a family dentist: “Having a dentist with whom you can consult for any problems and who will refer you to a specialist if necessary, and having received at least one dental checkup within the past year.” Thereafter, those with these conditions were referred to as the “FD group.”

### 2.3. Explanatory Variables

Regarding the explanatory variables, we set age, household income, employment status, marital status, child status, and residential municipality as individual socioeconomic factors, and the number of teeth, frequency of brushing teeth, and the habit of interdental cleaning as oral health status.

Participants’ ages were categorized into six groups: 20–29, 30–39, 40–49, 50–59 years, 60–69, and ≥70 years. Furthermore, the household incomes of the participants were categorized into six groups: <JPY 2000 K, JPY 2000 K–< 4000 K, JPY 4000 K–< 6000 K, JPY 6000 K–< 8000 K, ≥JPY 8000 K, and unknown. As of 2018, the average annual household income in Japan was JPY 5523 K, and the median value was JPY 4370 K (JPY 1 K = USD 7.5, June 2022) [30]. The employment status was categorized into five groups: regular employee, non-regular employee, homemaker, self-employed and others, and not working. Moreover, marital status was categorized as married or single. The participants’ child status was categorized as having children or not having children. Based on the Japanese municipal system, the municipalities where the participants lived were categorized into four groups: metropolis (ordinance-designated cities with a population of 500,000 or more, and 23 special wards of Tokyo), core cities (ordinance-designated cities with a population of 200,000 or more, excluding metropolises), cities (cities with a population of 50,000 or more, excluding metropolises and core cities), and towns and villages (small municipalities that do not meet city requirements).

The number of teeth of the participants was categorized into four groups: 0–9, 10–19, 20–27, and 28 or more teeth. Data on the number of teeth were obtained by questioning the participants. Although there were two ways to determine the number of teeth, a dentist’s examination or using a questionnaire, there was no significant difference between the two methods [31,32]. The participants’ frequency of brushing teeth was categorized into four groups: ≥three times daily, twice daily, once daily, and sometimes/no brushing. The habit of interdental cleaning was categorized as yes or no.

### 2.4. Statistical Analysis

First, we evaluated the descriptive statistics for each variable, where all variables were used as categorical data. Furthermore, we divided the study participants by gender and calculated the proportion of each of the FD groups. A chi-squared test was used to compare the proportion of the data.

Second, for each gender, we evaluated the relationship between the outcome variable (FD or non-FD group) and the explanatory variables (individual socioeconomic factors and oral health status) by cross-tabulation. For each item of each explanatory variable, we compared the proportions of the data using a chi-squared test.

Third, to analyze the characteristics of the FD group by gender, a multiple logistic regression analysis was conducted (FD group = 1, non-FD group = 0). To analyze the primary outcome variable of the FD group, the explanatory variables of individual socioeconomic factors (age, household income, employment status, marital status, child status, and residential municipality) and oral health status (the number of teeth, frequency of brushing teeth, and the habit of interdental cleaning) were explored. In Model 1, individual socioeconomic factors were used as the explanatory variables and, in Model 2, in addition to Model 1 factors, oral health status was added as an explanatory variable. In both models, the odds ratio (OR) and 95% confidence interval (95% CI) of each explanatory variable were calculated using a forced-entry model. Significance tests were employed to evaluate the fit of each model. Moreover, STATA version 14 (StataCorp LLC, College Station, TX, USA) was used for data management and statistical analysis. Statistical significance was set at *p* < 0.05.

### 2.5. Ethical Consideration

This study was approved by the Research Ethics Committee of the School of Dentistry, Health Sciences University of Hokkaido, before it was conducted (July 2021, #213). The survey participants were registered with Macromill, Inc. and agreed to the usage of their data for research. Furthermore, their personal information was protected by Macromill, Inc. [33]

## 3. Results

### 3.1. Characteristics of the Study Participants and Proportion of the FD Group by Gender

Table 1 shows the characteristics of the participants in this study. The total number of study participants was 3556, with a gender breakdown of 1708 men and 1848 women.

**Figure 1:**
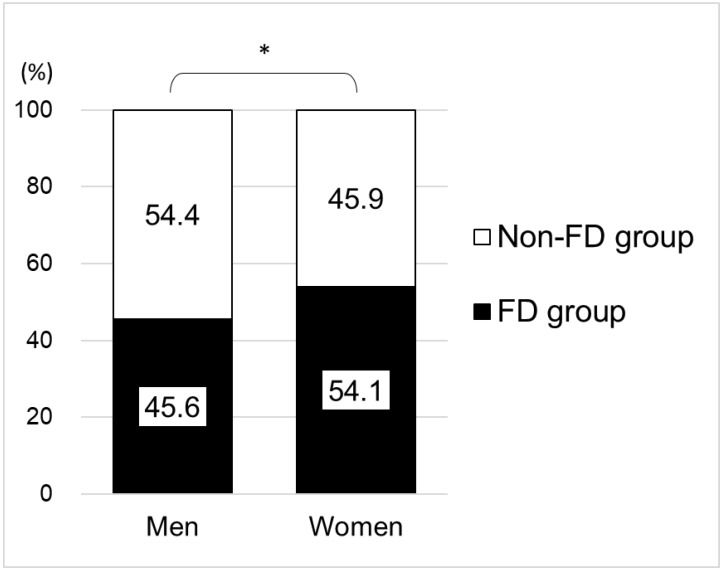
Proportion of the FD group by gender (total number: 3556; 1708 men, 1848 women). Note: Combined men and women: of 3556 participants, 1778 were in the FD group (50.0%); FD group = a group of those who regularly manage their oral health by having a family dentist, chi-squared test; * *p* < 0.001.

Figure 1 shows a comparison of the FD and non-FD groups by gender. Among the 1708 male study participants, 45.6% belonged to the FD group, while among the 1848 female study participants, 54.1% were in the FD group. Statistically significant differences were observed in the comparison of FD groups by gender (*p* < 0.001).

Of the 3556 study participants, 1778 were in the FD group (50.0%), combining men and women (not shown in Figure 1).

### 3.2. Relationship between Belonging to the FD Group or Not, and Characteristics of the Study Participants

Table 2 shows the relationship between belonging to the FD group or not (non-FD group), by gender, and the characteristics of the study participants. Among the male study participants, there were statistically significant differences in the age (χ^2^(5) = 71.58, *p* < 0.001), household income (χ^2^(5) = 27.54, *p* < 0.001), employment status (χ^2^(4) = 9.52, *p* = 0.049), marital status (χ^2^(1) = 35.91, *p* < 0.001), child status (χ^2^(1) = 21.23, *p* < 0.001), number of teeth (χ^2^(3) = 18.23, *p* < 0.001), frequency of brushing teeth (χ^2^(3) = 42.26, *p* < 0.001), and habit of interdental cleaning (χ^2^(1) = 192.85, *p* < 0.001). Among the female study participants, there were statistically significant differences in age (χ^2^(5) = 68.86, *p* < 0.001), household income (χ^2^(5) = 12.40, *p* = 0.030), marital status (χ^2^(1) = 13.23, *p* < 0.001), child status (χ^2^(1) = 18.62, *p* < 0.001), number of teeth (χ^2^(3) = 12.79, *p* = 0.005), frequency of brushing teeth (χ^2^(3) = 23.95, *p* < 0.001), and habit of interdental cleaning (χ^2^(1) = 202.56, *p* < 0.001).

### 3.3. Characteristics of Participants in the FD Group

Table 3 and Table 4 present the odds ratios in the multiple logistic regression analysis for the FD group in comparison to the non-FD group, adjusted for their relationship to the study participants’ characteristics (FD group = 1, non-FD group = 0).

Regarding the male study participants (Table 3), in the analysis of Model 1, those in the FD group mostly belonged to older age groups (60–69 years, OR: 1.46, 95% CI: 1.04–2.05; ≥70 years, OR: 2.50, 95% CI: 1.68–3.72), had higher household incomes (≥JPY 8000 K, OR: 1.58, 95% CI: 1.14–2.18), and fewer lived in rural areas (towns and villages, OR: 0.56, 95% CI: 0.36–0.87). Furthermore, in the analysis of Model 2, those in the FD group mostly belonged to older age groups (≥70 years, OR: 2.41, 95% CI: 1.56–3.71), had higher household incomes (≥JPY 8000 K, OR: 1.47, 95% CI: 1.05–2.08), fewer were living in rural areas (towns and villages, OR: 0.52, 95% CI: 0.32–0.83), a higher number were brushing their teeth three or more times daily (≥three times daily, OR: 1.60, 95% CI: 1.16–2.20), and were in the habit of interdental cleaning (OR: 3.66, 95% CI: 2.95–4.54).

Regarding the male study participants (Table 4), in the analysis of Model 1, those in the FD group were mostly belonged to the older age groups (60–69 years, OR: 1.67, 95% CI: 1.20–2.32; ≥70 years, OR: 2.01, 95% CI: 1.46–2.78), fewer were in the younger age groups (20–29 years, OR: 0.69, 95% CI: 0.48–0.99), and fewer with lower household incomes (<JPY 2000 K, OR: 0.61, 95% CI: 0.41–0.90). In the analysis of Model 2, those in the FD group mostly belonged to the older age groups (60–69 years, OR: 1.52, 95% CI: 1.07–2.16; ≥70 years, OR: 1.73, 95% CI: 1.22–2.46), fewer had lower household incomes (<JPY 2000 K, OR: 0.61, 95% CI: 0.40–0.92), and practiced habitual interdental cleaning (OR: 3.68, 95% CI: 2.96–4.57).

## 4. Discussion

### 4.1. Main Findings

We evaluated the proportion and characteristics of the FD group, characterized as those who regularly managed their oral health by having a family dentist. Our analysis showed that among the 3556 study participants, 45.6% of men and 54.1% of women belonged to the FD group. Compared to the men in the non-FD group, the majority of those in the FD group were in the older age groups, had higher household incomes, brushed their teeth three or more times daily, practiced habitual interdental cleaning, and fewer were living in rural areas. Regarding women, the majority were in the older age groups and practiced habitual interdental cleaning, while fewer had lower household incomes.

Therefore, our results suggest that despite Japan being a country with a public insurance coverage system for both men and women, having a family dentist is associated with disparities in individual socioeconomic factors, particularly age and household income.

### 4.2. Proportion of the FD Group

With regard to the status of having a family dentist, there are differences among countries. For example, a Canadian study shows 95% of the participants having a family dentist [20], a Saudi Arabian study shows 22% [22], and a South African study shows 3% [24]. However, since healthcare systems vary from country to country, it is impossible to simply compare these proportion values. On the other hand, in few Japanese studies, a survey of those aged 65 and older in some areas (Aichi area) indicated a proportion of 85% [18], and a survey of those aged 55–75 years in some areas (Akita area) indicated 88% [19]. Our study shows that 50% (gender breakdown: 45.6% men and 54.1% women) are in the FD group (Figure 1), and although there are differences from the above-mentioned previous studies [18,19], the following reasons are possible. First, in our study, we conducted a nationwide, web-based survey of randomly selected participants aged 20 years and older, using the quota sampling method, approximating the Japanese population. Although sample bias cannot be denied because the participants were selected from the registrants of the web-based survey company (see Section 4.6. for details), our study participants were very different from participants from a subset of the regions in previous studies [18,19]. Second, we clearly set “the definition of having a family dentist” in conducting the survey because there is no registration system for family dentists in Japan. Thus, we feared that not clarifying this definition would lead to confusion when study participants answered the survey. Therefore, there may be differences between the participants in our study and participants in previous studies regarding the meaning of having a family dentist.

In our study, the proportion of women in the FD group (54.1%) was higher than the proportion of men in the FD group (45.6%). It has been indicated that women are more aware and have better habits than men in terms of behaviors that protect oral health [34,35]. Therefore, this trend of gender differences strongly supports our observations.

### 4.3. Relationship between the FD Group and Individual Socioeconomic Factors

In the multiple logistic regression analysis results of Model 2 (men: Table 3; women: Table 4), among the men, the socioeconomic factors for the FD group were associated with older age groups, higher household incomes, and fewer rural residents. Meanwhile, among the women, there was an association with older age groups and having fewer lower household incomes.

With respect to age, Japanese government statistics reported that the older age groups had a higher prevalence of dental disease and visited the dentist more frequently than the younger age groups [36,37]. In addition, the older age groups were more financially well-off than the younger age groups [38], and consequently, this might have been one of the factors contributing to having a family dentist. In fact, since our study shows that the FD group is associated with household income for both men and women, this result supports the discussion above.

Higher household income is an important associated factor for enabling regular dental visits. This is a common observation in studies across several countries [4,6,34]. However, it is important to note that although Japan is believed to have fewer barriers to having dental visits due to household financial reasons than other countries because of its public health insurance, our study shows that household income is one of the associated factors for the FD group and is a barrier to access to dental utilization, and thus has a negative impact on oral health [4,6,34]. Hence, our results support the need for economic policy intervention (the details are described in the Section 4.5.).

Furthermore, with respect to residential location, several studies have shown that dental utilization is lower in rural areas than in urban areas [34]. However, it has been shown that there is little inequality in the geographic distribution of the number of dental clinics across the country in Japan, that is, there are fewer barriers to access to dental utilization in rural and urban areas [39]. In fact, in our study, there were fewer men in the FD group living in rural areas; however, no significant association was found among women. Therefore, our results may be caused by lower awareness of dental prevention among men in rural areas rather than because of differences in dental supply status by geographical location.

On the other hand, regarding employment, marital, and child statuses, no statistically significant associations were observed. These data were obtained from information on individual survey participants obtained from a web research company, and further detailed information was not used in this study. That is, if these data were subdivided further (e.g., classification by type of occupation; classification of marital status, including marital history; and classification of child status, including number of children), different results may have been obtained. Therefore, these results are difficult to determine from the present study alone and require further analysis.

### 4.4. Relationship between the FD Group and Oral Health Status Factors

In the multiple logistic regression analysis results of Model 2 (men: Table 3; women: Table 4), after adjusting for socioeconomic factors, as characteristics of the FD group, several male participants brushed their teeth at least three times a day and practiced habitual interdental cleaning. Similarly, many female participants practiced habitual interdental cleaning as well. Several previous reports have shown that having a family dentist is associated with trust in the dentist [15] and a higher patient satisfaction [23]. Moreover, interdental cleaning habits are an important strategy for preventing dental disease [40]. Altogether, those in the FD group had a higher awareness of prevention of dental diseases, which led to the habit of interdental cleaning. Hence, as shown by our results, it is possible that the FD group is more likely than the non-FD group to have good oral cleaning habits, particularly interdental cleaning, for both men and women.

On the other hand, with regard to the number of teeth, the results of the multiple logistic regression analysis (Table 3 and Table 4) show a marginally significant association with 20–27 teeth in women (OR: 1.62, 95% CI: 0.94–2.80, *p* = 0.082, reference: 0–9 teeth); otherwise, no statistically significant association was observed. This may be due to the fact that the FD group, both men and women, included many older adults, and these older participants tended to have fewer teeth than younger participants—by cross-tabulation (not shown in the figures and tables). Therefore, in a multiple logistic regression analysis adjusting for other factors, there was no significant association regarding the number of teeth.

### 4.5. Implications of the Study

In Japan, dental care is covered by public health insurance and is freely accessed at dental clinics all over the country [11]. It can be said that this is the realization of the UHC system advocated by the UN and WHO [9,10]. It is therefore thought that there might be fewer barriers to having a family dentist and managing oral health regularly, compared to other countries [4,5,6,7,8]. However, our study showed that the FD group was associated with individual socioeconomic factors, such as older age groups, higher household income, and rural residence for men, and older age groups and fewer lower household incomes for women. These socioeconomic factors, particularly household income, were difficult to resolve through individual efforts, and economic policy interventions can be considered. To motivate people to have a family dentist, it is necessary to develop interventions and measures, such as providing dental checkups with no out-of-pocket payment. Policymakers should plan dental policies without inequity, to protect oral health, with consideration of the people’s socioeconomic factors.

### 4.6. Limitations of the Study

This study had several limitations. First, the study participants were limited to registrants of the web-based survey company. Internet usage among the Japanese is increasing [41] and the web-based survey company used in our study had a large number of registered users [25]. We also used the quota sampling method to randomly select study participants from among these registered users. However, this sample is not reflective of the Japanese population. Therefore, it cannot be completely denied that sample bias may have occurred for the participants in this study. Second, this study was conducted as a cross-sectional survey using a web-based survey. Therefore, although we observed associations between the FD group and some individual socioeconomic factors and oral health behaviors, we could not determine the causal relationship between these factors. Third, although this study was conducted one and a half years after the COVID-19 pandemic began in Japan (March 2020), rather than immediately after, the possibility that the pandemic may have had an effect on the ability to have a family dentist cannot be disregarded. In particular, regarding the household income related to the FD group in this study, it was reported that income was affected by the COVID-19 pandemic [42], and therefore may have affected having a family dentist. Further detailed studies are required on the factors that influence having a family dentist, such as the causal relationships and the influence of the COVID-19 pandemic.

## 5. Conclusions

Using a nationwide web-based survey in Japan, we analyzed the proportion and characteristics of men and women in the FD group, characterized as those who regularly manage their oral health by having a family dentist. We observed that among the 3556 study participants, 45.6% men and 54.1% women were in the FD group. The FD group in men, compared to the non-FD group, had the following characteristics, including being in the older age groups, having higher household incomes, fewer were living in rural areas, having higher number of individuals who brush their teeth three or more times daily, and a habit of interdental cleaning. In women, the characteristics of the FD group included being in the older age groups, fewer with lower household incomes, and having a habit of interdental cleaning.

These results suggest that despite Japan being a country with a public insurance coverage system for both men and women, having a family dentist is associated with disparities in individual socioeconomic factors, particularly age and household income.

## Figures and Tables

**Table 1:** Characteristics of the study participants.

	Total		Men		Women	
Variable	Number	(%)	Number	(%)	Number	(%)
Total	3556	(100.0)	1708	(100.0)	1848	(100.0)
Age						
20–29 years	430	(12.1)	218	(12.8)	212	(11.4)
30–39 years	545	(15.0)	271	(15.9)	264	(14.3)
40–49 years	629	(17.7)	317	(18.5)	312	(16.9)
50–59 years	529	(14.9)	263	(15.4)	266	(14.4)
60–69 years	618	(17.4)	302	(17.7)	316	(17.1)
≥70 years	815	(22.9)	337	(19.7)	478	(25.9)
Household income						
<JPY 2000 K	299	(8.4)	114	(6.7)	185	(10.0)
JPY 2000 K–< 4000 K	850	(23.9)	427	(25.0)	423	(22.9)
JPY 4000 K–< 6000 K	703	(19.8)	371	(21.7)	332	(18.0)
JPY 6000 K–< 8000 K	455	(12.8)	241	(14.1)	214	(11.6)
≥JPY 8000 K	478	(13.4)	284	(16.6)	194	(10.5)
Unknown	771	(21.7)	271	(15.9)	500	(27.0)
Employment status						
Regular employee	1351	(38.0)	947	(55.4)	404	(21.9)
Non-regular employee	454	(12.8)	112	(6.6)	342	(18.5)
Homemaker	756	(21.3)	5	(0.3)	751	(40.6)
Self-employed and others	339	(9.5)	212	(12.4)	127	(6.8)
Not working	656	(18.4)	432	(25.3)	224	(12.1)
Marital status						
Married	2296	(64.6)	1073	(62.8)	1223	(66.2)
Single	1260	(35.4)	635	(37.2)	625	(33.8)
Child status						
With children	2233	(62.8)	958	(56.1)	1275	(69.0)
No children	1323	(37.2)	750	(43.9)	573	(31.0)
Municipalities						
Metropolis (pop 500,000+)	1242	(34.9)	597	(34.9)	645	(34.9)
Core cities (pop 200,000+)	685	(19.3)	337	(19.7)	348	(18.8)
Cities (pop 50,000+)	1417	(39.8)	669	(39.2)	748	(40.5)
Towns and villages	212	(6.0)	105	(6.2)	107	(5.8)
Number of teeth						
0–9	183	(5.2)	111	(6.5)	72	(3.9)
10–19	369	(10.4)	176	(10.3)	193	(10.4)
20–27	1392	(39.1)	673	(39.4)	719	(38.9)
≥28	1612	(45.3)	748	(43.8)	864	(46.8)
Frequency of brushing teeth						
≥Three times daily	928	(26.1)	328	(19.2)	600	(32.5)
Twice daily	1894	(53.3)	885	(51.8)	1009	(54.6)
Once daily	674	(18.9)	442	(25.9)	232	(12.5)
Sometimes/No brushing	60	(1.7)	53	(3.1)	7	(0.4)
Interdental cleaning						
Yes	2040	(57.6)	832	(49.1)	1208	(65.4)
No	1500	(42.4)	862	(50.9)	638	(34.6)

**Table 2:** Relationship between belonging to the FD or non-FD group and the characteristics of the participants.

	Men							Women						
Variable	Total Number	FD Group		Non-FD Group		χ^2^-Value	*p*-Value	Total Number	FD Group		Non-FD Group		Χ^2^ Value	*p*-Value
Total, *n* (%)	1708	779	(45.6)	929	(54.4)			1848	999	(54.1)	849	(45.9)		
Age, *n* (%)														
20–29 years	218	76	(34.9)	142	(65.1)	χ^2^(5) = 71.58	<0.001	212	84	(39.6)	128	(60.4)	χ^2^(5) = 68.86	<0.001
30–39 years	271	96	(35.4)	175	(64.6)			264	124	(47.0)	140	(53.0)		
40–49 years	317	133	(42.0)	184	(58.0)			312	160	(51.3)	152	(48.7)		
50–59 years	263	108	(41.1)	155	(58.9)			266	119	(44.7)	147	(55.3)		
60–69 years	302	152	(50.3)	150	(49.7)			316	198	(62.7)	118	(37.3)		
≥70 years	337	214	(63.5)	123	(36.5)			478	314	(65.7)	164	(34.3)		
Household income, *n* (%)														
<JPY 2000 K	114	42	(36.8)	72	(63.2)	χ^2^(5) = 27.54	<0.001	185	89	(48.1)	96	(51.9)	χ^2^(5) = 12.40	0.030
JPY 2000 K–<4000 K	427	212	(49.6)	215	(50.4)			423	233	(55.1)	190	(44.9)		
JPY 4000 K–<6000 K	371	165	(44.5)	206	(55.5)			332	194	(58.4)	138	(41.6)		
JPY 6000 K–<8000 K	241	113	(46.9)	128	(53.1)			214	121	(56.5)	93	(43.5)		
≥JPY 8000 K	284	153	(53.9)	131	(46.1)			194	115	(59.3)	79	(40.7)		
Unknown	271	94	(34.7)	177	(65.3)			500	247	(49.4)	253	(50.6)		
Employment status, *n* (%)														
Regular employee	947	415	(43.8)	532	(56.2)	χ^2^(4) = 9.52	0.049	404	194	(48.0)	210	(52.0)	χ^2^(4) = 13.48	0.090
Non-regular employee	112	45	(40.2)	67	(59.8)			342	188	(55.0)	154	(45.0)		
Homemaker	5	3	(60.0)	2	(40.0)			751	439	(58.5)	312	(41.5)		
Self-employed and others	212	93	(43.9)	119	(56.1)			127	64	(50.4)	63	(49.6)		
Not working	432	223	(51.6)	209	(48.4)			224	114	(50.9)	110	(49.1)		
Marital status, *n* (%)														
Married	1073	549	(51.2)	524	(48.8)	χ^2^(1) = 35.91	<0.001	1223	698	(57.1)	525	(42.9)	χ^2^(1) = 13.23	<0.001
Single	635	230	(36.2)	405	(63.8)			625	301	(48.2)	324	(51.8)		
Child status, *n* (%)														
With children	958	484	(50.5)	474	(49.5)	χ^2^(1) = 21.23	<0.001	1275	732	(57.4)	543	(42.6)	χ^2^(1) = 18.62	<0.001
No children	750	295	(39.3)	455	(60.7)			573	267	(46.6)	306	(53.4)		
Municipalities, *n* (%)														
Metropolis (pop 500,000+)	597	275	(46.1)	322	(53.9)	χ^2^(3) = 6.13	0.105	645	369	(57.2)	276	(42.8)	χ^2^(3) = 4.42	0.219
Core cities (pop 200,000+)	337	161	(47.8)	176	(52.2)			348	181	(52.0)	167	(48.0)		
Cities (pop 50,000+)	669	307	(45.9)	362	(54.1)			748	396	(52.9)	352	(47.1)		
Towns and villages	105	36	(34.3)	69	(65.7)			107	53	(49.5)	54	(50.5)		
Number of teeth, *n* (%)														
0–9	111	40	(36.0)	71	(64.0)	χ^2^(3) = 18.23	<0.001	72	28	(38.9)	44	(61.1)	χ^2^(3) = 12.79	0.005
10–19	176	98	(55.7)	78	(44.3)			193	111	(57.5)	82	(42.5)		
20–27	673	328	(48.7)	345	(51.3)			719	413	(57.4)	306	(42.6)		
≥28	748	313	(41.8)	435	(58.2)			864	447	(51.7)	417	(48.3)		
Frequency of brushing teeth, *n* (%)														
≥Three times daily	328	191	(58.2)	137	(41.8)	χ^2^(3) = 42.26	<0.001	600	368	(61.3)	232	(38.7)	χ^2^(3) = 23.95	<0.001
Twice daily	885	405	(45.8)	480	(54.2)			1009	520	(51.5)	489	(48.5)		
Once daily	442	172	(38.9)	270	(61.1)			232	110	(47.4)	122	(52.6)		
Sometimes/no brushing	53	11	(20.8)	42	(79.2)			7	1	(14.3)	6	(85.7)		
Interdental cleaning, *n* (%)														
Yes	832	524	(63.0)	308	(37.0)	χ^2^(1) = 192.85	<0.001	1208	798	(66.1)	410	(33.9)	χ^2^(1) = 202.56	<0.001
No	862	253	(29.4)	609	(70.6)			638	200	(31.3)	438	(68.7)		

Note: FD group = a group of those who regularly manage their oral health by having a family dentist; chi-squared test.

**Table 3:** Characteristics of the male study participants in the FD group.

	Model 1			Model 2		
Variable	OR	95% CI	*p* Value	OR	95% CI	*p*-Value
Age						
20–29 years	0.90	(0.61–1.32)	0.582	1.03	(0.69–1.55)	0.880
30–39 years	0.79	(0.57–1.12)	0.185	0.84	(0.58–1.20)	0.328
40–49 years	1.00	Reference		1.00	Reference	
50–59 years	0.92	(0.66–1.29)	0.638	0.99	(0.69–1.42)	0.947
60–69 years	1.46	(1.04–2.05)	0.030	1.33	(0.92–1.92)	0.124
≥70 years	2.50	(1.68–3.72)	<0.001	2.41	(1.56–3.71)	<0.001
Household income						
<JPY 2000 K	0.74	(0.46–1.18)	0.204	0.95	(0.57–1.59)	0.835
JPY 2000 K–<4000 K	1.10	(0.82–1.48)	0.530	1.12	(0.81–1.53)	0.495
JPY 4000 K–<6000 K	1.00	Reference		1.00	Reference	
JPY 6000 K–<8000 K	1.15	(0.82–1.61)	0.416	1.09	(0.76–1.55)	0.637
≥JPY 8000 K	1.58	(1.14–2.18)	0.006	1.47	(1.05–2.08)	0.027
Unknown	0.76	(0.54–1.07)	0.119	0.80	(0.56–1.15)	0.230
Employment status						
Regular employee	1.00	Reference		1.00	Reference	
Non–regular employee	0.86	(0.56–1.34)	0.516	0.85	(0.53–1.35)	0.483
Homemaker	1.61	(0.25–10.56)	0.619	1.49	(0.20–11.26)	0.698
Self–employed and others	0.83	(0.59–1.15)	0.260	0.82	(0.57–1.16)	0.258
Not working	0.96	(0.70–1.32)	0.808	0.92	(0.65–1.30)	0.633
Marital status						
Married	1.36	(1.00–1.85)	0.052	1.32	(0.95–1.83)	0.099
Single	1.00	Reference			Reference	
Child status						
With children	0.90	(0.67–1.20)	0.462	0.88	(0.65–1.19)	0.409
No children	1.00	Reference			Reference	
Municipalities						
Metropolis (pop 500,000+)	1.00	(0.79–1.26)	0.980	0.90	(0.70–1.15)	0.388
Core cities (pop 200,000+)	1.08	(0.82–1.42)	0.588	1.08	(0.81–1.44)	0.614
Cities (pop 50,000+)	1.00	Reference		1.00	Reference	
Towns and villages	0.56	(0.36–0.87)	0.010	0.52	(0.32–0.83)	0.007
Number of teeth						
0–9				1.00	Reference	
10–19				1.57	(0.90–2.72)	0.110
20–27				1.21	(0.75–1.93)	0.436
≥28				1.07	(0.66–1.72)	0.794
Frequency of brushing teeth						
≥Three times daily				1.60	(1.16–2.20)	0.004
Twice daily				1.27	(0.99–1.64)	0.064
Once daily				1.00	Reference	
Sometimes/no brushing				0.55	(0.24–1.30)	0.176
Interdental cleaning						
Yes				3.66	(2.95–4.54)	<0.001
No				1.00	Reference	

Note: FD group = a group of those who regularly manage their oral health by having a family dentist; OR = odds ratio; 95%CI, 95% confidence interval. Model 1: Number of observations = 1708, χ^2^(19) = 113.10, Log likelihood = −1120.75, *p* < 0.001; Model 2: Number of observations = 1694, χ^2^(26) = 296.35, Log likelihood = −1020.23, *p* < 0.001.

**Table 4:** Characteristics of the female participants in the FD group.

	Model 1			Model 2		
Variable	OR	95% CI	*p* Value	OR	95% CI	*p*-Value
Age						
20–29 years	0.69	(0.48–0.99)	0.045	0.85	(0.57–1.25)	0.410
30–39 years	0.84	(0.60–1.17)	0.297	0.93	(0.65–1.32)	0.678
40–49 years	1.00	Reference		1.00	Reference	
50–59 years	0.76	(0.55–1.07)	0.112	0.71	(0.50–1.00)	0.052
60–69 years	1.67	(1.20–2.32)	0.002	1.52	(1.07–2.16)	0.020
≥70 years	2.01	(1.46–2.78)	<0.001	1.73	(1.22–2.46)	0.002
Household income						
<JPY 2000 K	0.61	(0.41–0.90)	0.014	0.61	(0.40–0.92)	0.019
JPY 2000 K–<4000 K	0.81	(0.60–1.10)	0.174	0.79	(0.57–1.09)	0.147
JPY 4000 K–<6000 K	1.00	Reference		1.00	Reference	
JPY 6000 K–<8000 K	0.96	(0.67–1.38)	0.838	0.96	(0.66–1.40)	0.818
≥JPY 8000 K	1.07	(0.74–1.55)	0.724	0.91	(0.62–1.35)	0.649
Unknown	0.79	(0.59–1.05)	0.106	0.77	(0.57–1.05)	0.094
Employment status						
Regular employee	1.00	Reference		1.00	Reference	
Non-regular employee	1.12	(0.82–1.53)	0.466	1.16	(0.83–1.61)	0.385
Homemaker	0.93	(0.70–1.26)	0.654	0.98	(0.72–1.34)	0.899
Self-employed and others	0.81	(0.53–1.23)	0.321	0.91	(0.58–1.43)	0.691
Not working	0.86	(0.59–1.24)	0.408	0.86	(0.58–1.27)	0.440
Marital status						
Married	1.15	(0.89–1.50)	0.285	1.08	(0.82–1.42)	0.583
Single	1.00	Reference			Reference	
Child status						
With children	1.08	(0.85–1.38)	0.529	1.10	(0.86–1.42)	0.444
No children	1.00	Reference			Reference	
Municipalities						
Metropolis (pop 500,000+)	1.19	(0.96–1.49)	0.115	1.07	(0.85–1.35)	0.560
Core cities (pop 200,000+)	0.96	(0.74–1.25)	0.769	0.90	(0.68–1.18)	0.440
Cities (pop 50,000+)	1.00	Reference		1.00	Reference	
Towns and villages	0.86	(0.57–1.31)	0.492	0.87	(0.56–1.35)	0.524
Number of teeth						
0–9				1.00	Reference	
10–19				1.46	(0.80–2.67)	0.216
20–27				1.62	(0.94–2.80)	0.082
≥28				1.46	(0.85–2.51)	0.175
Frequency of brushing teeth						
≥Three times daily				1.21	(0.86–1.69)	0.275
Twice daily				0.91	(0.67–1.25)	0.577
Once daily				1.00	Reference	
Sometimes/no brushing				1.00	(omitted)	
Interdental cleaning						
Yes				3.68	(2.96–4.57)	<0.001
No				1.00	Reference	

Note: FD group = a group of those who regularly manage their oral health by having a family dentist; OR = odds ratio; 95% CI = 95% confidence interval. Model 1: Number of observations = 1848, χ^2^(19) = 96.55, Log likelihood = −1226.57, *p* < 0.001; Model 2: Number of observations = 1841, χ^2^(25) = 268.78, Log likelihood = −1135.16, *p* < 0.001.

## Data Availability

Data cannot be shared publicly, because no informed consent was given by the participants for open data sharing.
